# Antioxidant Activity Modulated by Polyphenol Contents in Apple and Leaves during Fruit Development and Ripening

**DOI:** 10.3390/antiox9070567

**Published:** 2020-07-01

**Authors:** Aneta Wojdyło, Jan Oszmiański

**Affiliations:** Department of Fruit, Vegetable and Plant Nutraceutical Technology, Wrocław University of Environmental and Life Sciences, 37 Chełmońskiego Str., 51-630 Wrocław, Poland; jan.oszmianski@upwr.edu.pl

**Keywords:** *Malus domestica*, antioxidants, polyphenols, procyanidins, LC–MS, HPLC

## Abstract

Apples (*Malus domestica* Borkh) are of particular interest for their high content of healthy phytochemicals. This study investigated the antioxidant activity and polyphenolic compounds of extracts from the fruits from Ozark Gold, Starkinson and Kosztela cultivars and additionally leaves from Ozark Gold cv. Phenolic compounds were identified and quantified by liquid chromatography- mass spectrometer (LC–MS) and high pressure liquid chromatography (HPLC). The samples were collected during fruit development at 60, 80, 130 and 145 days after full bloom. The concentration of apple phenolics was highest early in the season and decreased during fruit development. The leaf phenolics had a more steady level during all the period of collection than the fruits. Among the five groups of analyzed polyphenols, the procyanidins predominated in apple fruits and dihydrochalcones in leaves. The concentration of polyphenols decreased from 39.15, 5.97 and 33.39 g/kg dw (collected at 60 days after full bloom) to 14.22, 2.05 and 9.31 g/kg dw (collected at 145 days after full bloom) for apples Ozark Gold, Starkinson, Kosztela, respectively. The leaves characterized a much higher content of polyphenolic compounds and antioxidant capacity than unripe fruits. Antioxidant capacity measured by 2,2-azinobis-(3-ethyl-benzothiazoline-6-sulfonic acid (ABTS) and 2,2-diphenyl-picrylhydrazyl (DPPH) methods was higher when the apple was unripe and the leaves were young. The results indicate that, unripe fruits and leaves are very good raw material for polyphenol nutraceutical production with high antioxidant potential.

## 1. Introduction

In recent years, the secondary metabolites, which occur abundantly in plant foods, have been found by human nutritionists to be beneficial components of functional food. Their positive effects on human health are now widely accepted [1,2].

Apple fruit (*Malus domestica* Borkh) are one of the major fruits available on the market in the world and the consumption of this fruit is common in many countries. Therefore, this fruit is one of the major sources of polyphenol compounds [3] which play an important role for the protection of humans in many civilization diseases such as type-2 diabetes, lung cancer, ischemic heart disease, asthma or thrombotic stroke. In addition, eating apples was also associated with increased lung function and increased weight loss [4,5,6,7].

Generally, the major polyphenolic groups, found in various apple varieties, are hydroxycinnamic acids, mainly chlorogenic acid; flavan-3-ols as derivatives of (+)-catechin or (−)-epicatechin as monomers; dimers, oligomers or polymers of procyanidins, and dihydrochalcones, mainly connected with glucose and xyloglucose; flavonols, mainly as derivatives of quercetin and keampferol associated by galactose, glucose, rhamnose, arabinose or xylose; and then finally the last classed are anthocyanins [8,9,10,11,12,13].

The chemical complexity and the variations of the apple’s phenolic profile are caused by the growth period, growing season, geographical location, and most importantly, genetic variation [14,15]. Less is known about the variation of polyphenol concentrations during apple growth. Mosel and Herrmann [16] found large quantities of hydroxycinnamic acids and catechins in early fruits, and a decrease in their concentrations during growth. Renard et al. [17] was describing the changes of the concentrations of apple flavonoids (flavan-3-ols, dihydrochalcones and flavonols) in two table cultivars and two cider cultivars, collected during fruit growth and maturation. It was found that polyphenol accumulation in the apple flesh occurred early during the fruit life and that the evolution of the concentrations during fruit growth and maturation was essentially due to the dilution of an initially accumulated store. 

It is clear that dessert apples and apple products containing these bioactive components are contributing to the maintenance of human health and to the prevention of disease. Moreover, apple pomace, unripe fruits, and other plant organs also serve as the raw material for obtaining purified phenolic preparations. Thus, the polyphenol compounds from these plants should be contemplated as highly valuable sources that may be used as functional food ingredients and as natural antioxidants to replace some synthetic equivalents which nowadays are less accepted by the consumer and food industry, having experienced growing rejection.

Natural antioxidants occur in all parts of plants. Leaves have traditionally been used in various cultures, such as European and Asian, as food or to improve the appearance (color, odor, and flavor) and nutritional value of meals. Sage, mint and nettle leaves have been used for decades. The edible leaves of cabbage, spinach, etc. are widely consumed and are popular components of standard diets in many parts of the world. The leaves are served as salads, used to prepare cakes, beverages or teas, or consumed as a side dish. It is known that edible leaves (herbs and vegetables) are rich in many natural antioxidants including flavonoids, phenolic acids, tri- and tetraterpenes, iridoids and many other bioactive compounds, and they constitute an important source of bioactive substances in the human diet. Oliveira et al. [18] have shown that *Cydonia oblonga* leaves are rich sources of phenolic content, varying from 4.9 to 16.5 g/kg dry matter with relatively high contents of kaempferol derivatives than other parts of fruits as the pulps, peels, and seeds. Additionally, they postulated that these leaves can be used as a good and cheap source of bioactive constituents. Several other studies have previously presented that the leaves of barley, Prunus, and Eucalytus or the leaves of berry crops possess a characteristic high content of bioactive compounds, higher than in fruits, and present a high antioxidant power [19,20]. To our knowledge, little information is available on the antioxidant capacities in the leaves and fruits of apples at different developmental stages. Ridgway et al. [21] called the apple a “new agrochemical crop”. They reviewed all the prospects which are founded on the high amounts of phloridzin produced by apple leaves and twigs. 

Leaves’ extracts are presently gaining greater attention as phytochemicals and have relevance in the context of nutraceuticals. The polyphenols extracted from other raw materials, other than ripe apple tissues, such as unripe fruits or leaves, serve as the raw material for obtaining purified phenolic preparation added to foods as nutraceuticals. The purpose of this work was to highlight the potential of selected by-products as a source of functional compounds.

Therefore, the main objective of the present study was to establish the antioxidant activity modulated by phenolic content (LC–MS and HPLC) in unripe fruits during apple development. The investigation on apple leaves as a potential source of polyphenolics with a high antioxidant activity power was also included and has not been reported by the other authors. 

## 2. Materials and Methods

### 2.1. Plant Material and Sample Preparation

Apple fruits Ozark Gold, Starkinson, Kosztela cvs. and the leaves of Ozark Gold cv. were grown in Zybiszów near Wrocław (51°06′N 16°92′E; Poland) and harvested on 20 July, 10 August, 4, 19 September, and the 20 October in 2018 (every 3 weeks). After harvest, the whole apples (all fruits (five apples) and leaves (ten leaves) were from the same part of tree) and the leaves were cut directly in liquid nitrogen, and freeze-dried (24 h; Alpha 1-4, Christ, Osterode am Harz, Germany). The homogeneous powders were obtained by crushing the dried tissues with the use of a closed laboratory mill (IKA 11A; Staufen, Germany) to avoid hydration, and then analyzed. 

### 2.2. Extraction Procedure

Ground dry plant materials (1 g) were weighed into a test tube and slightly stirred with 20 mL of 80% aqueous methanol with 1% of HCl. Then, the samples were sonicated for 15 min and left at 4 °C in darkness for 24 h. After this period, the samples were again sonicated, then centrifuged for 10 min (20,878× *g*), and the supernatants were collected at 4 °C to be used within 24 h. Before the analysis by LC–MS and HPLC, the extract was filtered by a 0.20 μm hydrophilic polytetrafluoroethene (PTFE) membrane (Millex Simplicity Filter; Merck, Darmstadt, Germany). For the determination of antioxidant activities, the same protocol as that described above was used, but a methanol/water (80:20, *v*/*v*) with 1% hydrochloric acid mixture was used for extraction.

### 2.3. Identification of Polyphenols by LC–MS Method

The identification of the polyphenols in the apple extracts and leaves was conducted using a Symmetry C18 column (5 μm × 150 × 4.6 mm; Waters Corp., Milford, MA, USA) LC-MS system. The chromatographic system consisting of a gradient HPLC separation module type 2690 (Waters Corp., Milford, MA, USA) co-worked with a 996 diode array ultraviolet/visible absorbance detector (Waters Corp., Milford, MA, USA), and a quadrupole ion tunnel mass spectrometer (Quattro Ultima, Micromass Ltd., Manchester, UK) equipped with a electrospray ionization (ESI) source. The separation was provided for 51 min at 20 °C with a flow rate of 0.5 mL/min. The mobile phase was composed of solvent A (1.0% formic acid in water, *v*/*v*) and solvent B (100% of acetonitrile). Elution was as follows: 95% A (0–1 min); from 95 to 0% A (1–41 min); 0% A (42–51 min) for column washing, and then reconditioning for the next 9 min. The source voltage was 0.1 and 3 kV for positive and negative ionization, respectively. The capillary temperature was 300 °C, the sheath and auxiliary gas were 50 and 5 units, respectively. The analysis was carried out using a full scan, and a retention time corresponding to the standards of chlorogenic acid, *p*-coumaric acid, quercetin-3-*O*-glucoside, -3-*O*-galactoside, -3-*O*-arabinoside, phloretin-2′-*O*-glucoside, (+)-catechin, (−)-epicatechin, procyanidin B1, B2 and C1, and data-dependent MS scanning from *m/z* 100 to 1000. 

### 2.4. Quantification of Polyphenols by HPLC Method

The analysis of flavan-3-ols and dihydrochalcones at 280 nm, phenolic acid at 320 nm, flavonols at 360 nm and anthocyanin at 520 nm were carried out on a liquid chromatography model L-7455 with a quaternary pump model L-7100 equipped with a Multisolvent Delivery System model D-7000 HSM cooperated with a diode array detector (DAD), and an autosampler model L-7200 (Merck-Hitachi, Tokyo, Japan). The separation was performed on a Synergi Fusion RP-80A (150 × 4.6 mm, 4 μm; Phenomenex Torrance, California, USA) column at 30 °C and the flow rate was 1.0 mL/min. The mobile phase was composed of solvent A (2.5% formic acid in water, *v*/*v*) and solvent B (100% of acetonitrile). Elution was the same as described in Section 2.3. The retention times and spectra were compared with those of pure standards within 200–600 nm. The calibration curves were made from (−)-epicatechin, (+)-catechin, procyanidin B1, B2 and C1, chlorogenic acid, *p*-coumaric acid, phloretin-2′-*O*-glucoside, quercetin-3-*O*-glucoside, -3-*O*-galactoside, and -3-*O*-arabinoside as standards. Thus, phloretin-2′-xyloglucoside was quantified as phloretin-2′-*O*-glucoside, and *p*-coumaroylquinic acid was quantified as *p*-coumaric acid. All the samples were analyzed in triplicate and the results were expressed as g per kg dry weight (dw).

### 2.5. Polymeric Procyanidins Analysis by Thiolysis Method

The analysis of polymeric procyanidin fractions was performed by using an high pressure liquid chromatography—fluorescence detector (HPLC-FL) system (Waters Corporation; Milford, Michigan, USA). The analysis was performed as already described by Guyot et al. [22]. The thiolysis products were separated on a Merck Purospher RP 18 end-capped column (250 × 4 mm, 5 µm; Merck, Darmstadt, Germany) with solvent A as 2.5% of acetic acid and solvent B as acetonitrile. The run was set up with the following gradient: 0 min 3%, 0.5–5.0 min up to 9% B, 5.1–15.0 up to 16% B, and 15–45 min up to 50% B followed by 10 min for re-equilibration. The flow rate was 1 mL/min, the sample injection volume was 20 µL, the column temperature was 30 °C and the fluorescence was recorded at the excitation and emission wavelengths at 278 and 360 nm, respectively. The calibration curves (*R*^2^: 0.9989–0.9998) for quantification were made from procyanidin B2, (+)–catechin and (−)-epicatechin. The average degree of polymerization was calculated as the molar ratio of all flavan-3-ol units to the terminal units of (−)-epicatechin and (+)-catechin. All samples were analyzed in triplicate and the results expressed as g per kg dw.

### 2.6. Antioxidant Activities Measurements as 2,2-azinobis-(3-ethyl-benzothiazoline-6-sulfonic acid (ABTS) and 2,2-diphenyl-picrylhydrazyl (DPPH) Assay

The 2,2-diphenyl-picrylhydrazyl (DPPH) radical-scavenging activity was estimated according to the method of Yen et al. [23]. The radical stock solution of DPPH (100 µM) was prepared by dissolved ethanol at 96%. The 1 mL of sample extracts, 1 mL of DPPH solution and 3 mL of ethanol were mixed in cuvette and were allowed to stand at room temperature in the dark for 10 min. Absorbance was measured at 517 nm exactly after 10 min. 

The free-radical-scavenging activity with 2,2-azinobis-(3-ethyl-benzothiazoline-6-sulfonic acid (ABTS radical cation) was determined as reported by Re et al. [24]. The radical ABTS was dissolved in water to a 7 μM concentration and was produced by reacting with 2.45 μM potassium persulfate and allowed to stand for 12–16 h in the dark at room temperature. Prior to the assay, the ABTS^•+^ solution was diluted with redistilled water to achieve an absorbance at 734 nm of about 0.700 ± 0.02. The 3.0 mL of the diluted ABTS^•+^ solution was mixed with 30 μL of the sample extracts in the cuvette, and then exactly 6 min after the initial mixing, the absorbance was read. 

All the determinations (*n* = 3) were performed using a Shimadzu UV–vis 2401 PC spectrophotometer (Tokyo, Japan). All the results were expressed in mM Trolox per g of dry weight (dw) extract.

### 2.7. Statistical Analysis

The statistical analysis was conducted using Statistica version 6.0 (StatSoft, Kraków, Poland). Significant differences (*p* ≤ 0.05) between the average responses were evaluated by using one-way ANOVA with the Duncan test.

## 3. Results and Discussion

### 3.1. Qualitative Analysis of Polyphenol Compounds

**Figure 1:**
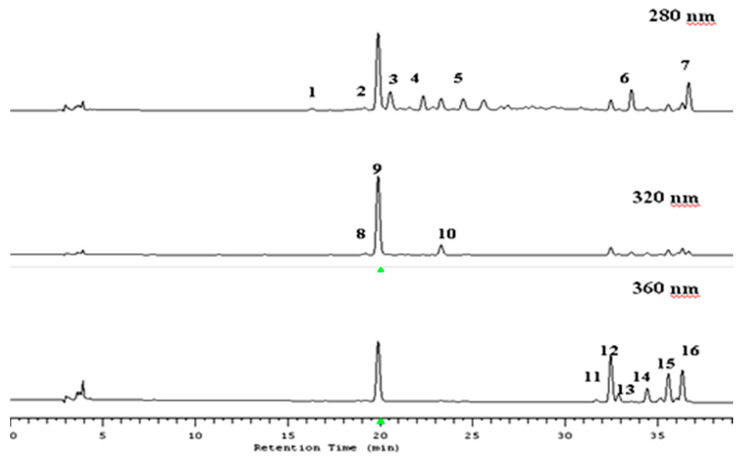
HPLC chromatograms of the apple extract at 280, 320 and 360 nm. 1—procyanidins B1; 2—(+)-catechin; 3—procyanidins B2; 4—(−)-epicatechin; 5—procyanidins C1; 6—phloretin 2′-xyloglucose; 7—phloretin 2′-glucose; 8—caffeic acid hexose conjugate; 9—chlorogenic acid; 10—*p*-coumaroyloquinic acid; 11—quercetin 3-*O*-rutinoside; 12—quercetin-3-*O*-galactoside; 13—quercetin-3-*O*-glucoside; 14—quercetin-3-*O*-arabinoside; 15—quercetin-3-*O*-xyloside; and 16—quercetin-3-*O*-rhamnoside.

As an initial step, the samples were analyzed by HPLC–MS. The typical HPLC traces are illustrated in Figure 1 and the mass spectral data obtained are summarized in Table 1. 

Five flavan-3-ols were detected: (+)-catechin, (−)-epicatechin, and procyanidin B1, B2, and C1. Peaks **1** and **3** (*λ*_max_ = 275 nm) are procyanidin B1 and B2 with *R*_t_ at 16.8 and 20.8 min, respectively, which had a (M − H)^−^ at *m*/*z* 577. Peaks **2** and **4** (*R*_t_ = 19.5 and 22.4 min) also had a *λ*_max_ of 275 nm and a mass spectrum with an *m*/*z* of 289, indicating the existence of a (+)-catechin and (−)-epicatechin, respectively. Peak **5** (*R*_t_ = 24.5 min, *λ*_max_ = 275 nm), with a characteristic highest molecular mass of flavan-3-ol compounds at *m*/*z* 865, is procyanidin C1.

Two dihydrochalcones were detected, namely phloretin-2′-*O*-xyloglucoside and phloretin-2′-*O*-glucoside. Peak **6** (*R*_t_ = 34.0 min, *λ*_max_ = 285nm), with a (M − H)^−^ at *m*/*z* 567 is phloretin-2′-*O*-xyloglucoside. Peak **7** (*R*_t_ = 36.8 min, *λ*_max_ = 285 nm) produced a (M − H)^−^ at *m*/*z* 435. This compounds’ pattern corresponds to phloretin-2′-*O*-glucoside.

Three hydroxycinnamates were detected, namely *p*-coumaroylquinic acid, 5-*O*-caffeoylquinic acid (chlorogenic acid) and caffeic acid hexose conjugate. Peak **8** (*R*_t_ = 23.5 min, *λ*_max_ = 305 nm) which had a (M – H)^−^ at *m/z* 337 was identified as *p-*coumaroylquinic acid. Peak **9** (*R*_t_ = 19.9 min, *λ*_max_ = 320) is 5-*O*-caffeoylquinic acid (as chlorogenic acid) and had characteristic mass spectral data as it produced a (M – H)^−^ at *m*/*z* 353. Peak **10** (*R*_t_ = 19.1 min, *λ*_max_ = 305 nm) had the mass spectral fragmentation pattern characteristic of caffeic acid hexose conjugate.

Four flavonols were conjugated with the glycosidic unit as -galactoside, -glucoside, -rhamnoside, -rutinoside, -arabinoside and -xyloside. Mass spectra allowed the identification of aglycone with a characteristic fragmentation *m/z* of 301 as a quercetin moiety. Peak **11** (*R*_t_ = 31.6 min, *λ*_max_ = 350 nm) suggested the presence of a quercetin-3-*O*-rutinoside, and this identification was confirmed by the mass spectral data which revealed a (M − H)^−^ at *m*/*z* 609. Both peaks **12** and **13** (*R*_t_ = 32.4 and 32.9 min) had an (M − H)^−^ at *m*/*z* 463 and the fragmentation yielded a quercetin moiety (*m*/*z* 301) with the characteristic loss of *m/z* 162, which indicates the cleavage of a hexose group as a glucoside or galactoside. These compounds were additionally identified as quercetin-3-*O*-galactoside (peak **12**) and quercetin-3-*O*-glucoside (peak **13**) after comparison to the standards’ compounds. A similar situation was found for quercetin-3-*O*-arabinoside (peak **14**) and quercetin-3-*O*-xyloside (peak **15**), as both had the same *m/z* 433 but had different retention times (*R*_t_ = 34.3 and 35.5 min, respectively). Peak **16** (*R*_t_ = 36.4 min, *λ*_max_ = 345 nm), which produced a (M − H)^−^ at *m*/*z* 447 and a fragment at *m*/*z* 301, was identified as quercetin-3-*O*-rhamnoside, which has been reported previously in apples [25].

### 3.2. Quantification Phenolic Compounds in Apple Fruits and Leaves

The distribution of the polyphenol constituents of the apple fruits and leaves under study are presented in Table 2 and Table 3. A total of 16 polyphenolic compounds belonging to five major polyphenolic groups were quantified by the HPLC method. The concentration of polyphenolic compounds changed significantly (*p* < 0.05) during the fruit development.

Polymeric procyanidins were also investigated after the direct thiolysis of the freeze-dried samples. Among the five groups, procyanidins and their monomer catechins were the most predominant phenolic group in apple fruits, however, to date little is known about procyanidins. In this study, it was observed that they decreased significantly (*p* < 0.05) during fruit development. The total procyanidin content ranged from 35.0, 5.01 and 20.72 g kg^−1^ dw in unripe fruits (60 days after full bloom) to 11.80, 1.68 and 5.30 g kg^−1^ dw in ripe fruits (145 days after full bloom). The highest changes of catechins with time were found in fruits of Kosztela cv., which decreased from 3.11 to 0.68 g kg^−1^ dw (Table 2). A higher concentration of proanthocyanidins in young fruit was also reported by Burda et al. [26]. The content of polyphenols, like proanthocyanidins, depends on cultivars and the growing season [10,27].

The hydroxycinnamic acids were the second most abundant group in the fruits and ranged during fruit development from 1.2 g kg^−1^ dw in unripe fruits (60 days after full bloom) to 0.3 g kg^−1^ dw in ripe fruits (145 days after full bloom) for Ozark Gold, from 0.34 to 0.05 g kg^−1^ dw for Starkinson and from 6.78 to 1.68 g kg^−1^ dw for Kosztela cvs., respectively. Chlorogenic acid was predominant phenolic acid in *Malus* unripe and ripening fruits compared to other phenolic acids.

During the growth and maturation of fruit, the dihydrochalcones of apples also decreased markedly. The concentration of phloretin-2′-*O*-glucoside and phloretin-2′-*O*-xyloglucoside are highest in July and fell sharply in October from 0.4 g kg^−1^ dw to 0.1 g kg^−1^ dw and from 0.3 g kg^−1^ dw to 0.1 g kg^−1^ dw for Ozark Gold cv., respectively. The same tendency was evaluated for rest analyzed cvs.

However, the variation of flavonols during fruit development was different than of other phenolics. The flavonols showed fluctuations during the investigated season. Mayr et al. [28] also reported that maximal flavonol concentrations were obtained during the third month after full bloom. Similar results were obtained for apples, in accordance with literature results. Renard et al. [17] have recently reported that the concentrations of flavonoids (flavan-3-ols, dihydrochalcones, and flavonols) in the fruit flesh of two table and two cider apple cultivars decreased sharply between 35 and 100 days after flowering.

Apple fruits such as Ozark Gold and Starkinson cvs. contain anthocyanin, mainly cyanidin 3-*O*-glucoside, however the content was below 0.3 and 0.5 g kg^−1^ dw, respectively, and it was observed that full ripe fruits contained the highest content of this compound. This is natural because the apple skin of some cvs. possesses characteristic red color.

Takos et al. [29] also reported a decrease in procyanidin concentrations and a degree of polymerization in the apple skin during early growth and maturation. The evolution of hydroxycinnamic acids concentrations were similar to those observed above; there was a continuous decrease in the concentration, faster at the earlier stages, then a relative stabilization, and the decrease appeared slower. The sum of the phenolic compounds determined by HPLC in our Ozark Gold cv. was distributed in a wide concentration range depending on the period after full blooming. Unripe apples (60 days after full bloom) had 2.7-fold more total polyphenols than ripe apples (145 days after full bloom). The same tendency was observed for the rest of the analyzed samples over the whole period. In blackberries and strawberries, the total phenolic content decreased significantly as the fruit matured from the green to the ripe stage [30]. Nuncio-Jáuregui et al. [31] presented that the content of ellagic acid derivatives in thinning pomegranate fruits were 3–19 times higher than those found in ripe fruits. In recent decades and still today, Poland is one of the biggest European apple producers [27]. Thinning is a routine farming practice, which takes place at an immature stage of the fruits, and consists of removing part of the fruits to benefit the development and quality of the remaining fruits [32]. This practice is very often used in Spanish farming when carried out for pomegranate fruits. During this operation, even among 7–15 kg per tree could be removed [32]. The fruits that remain in the tree experience significant changes in their physicochemical and phenolic compounds as well as antioxidant properties [33,34].

Table 3 shows the phenolic composition of the leaves as influenced by their age (the leaves and fruits were picked up at the same time). The concentrations in the leaves were 3.8 times those in the fruits at the earliest date, and 11.3 times those in the fruits at the latest date, all due to the decrease in concentrations compared to the Ozark Gold cv. fruits. The biggest qualitative difference in the polyphenolic composition between the fruits and leaves was in the dihydrochalcone and the flavonol concentration (Table 2 and Table 3). 

The dihydrochalcone phloretin-2′-*O*-glucoside in dry leaves was in a very high concentration: more than 11% of dw.

Nowadays, this molecule is used as a pharmaceutical component in the treatment of hypoglycemia because the principal action is producing renal glycosuria and blocking glucose transportation [35]. Additionally, the derivatives of phloretin-2′-*O*-glucoside are good inhibitors of lipid peroxidation, growth of human colon cancer cells, bone loss, and the enhancement of memory [36,37]. However, because commercial phloretin-2′-*O*-glucoside is currently produced from the root bark of apple trees, which can only be harvested once in the plant’s life cycle, its production cost is very high and thus its uses are seriously limited. Therefore, the leaves were found to be a potentially new substitute for the root bark of apple trees as a rich and cheaper source of phloretin-2′-*O*-glucoside production. 

Furthermore, the quercetin glycosides are also present in high concentrations in apple leaves. Additionally, in apple leaves, besides the quercetin derivatives typically of apple fruit, quercetin-3-*O*-rutinoside was also evaluated. During leaf maturation, the total concentration of -3-*O*-galactoside, -3-*O*-rhamnoside, -3-*O*-rutinoside, -3-*O*-glucoside, -3-*O*-arabinoside, and -3-*O*-xyloside remain almost at a constant level and range from 1.85% to 2.56% of dry weight. We found that apple leaves are a much better source of these compounds than apple fruits, especially after the fruit full ripens.

Liaudanskas et al. [38] postulated that besides phloridzin, quercitrin was the major compound among quercetin glycosides in the ethanol extracts of the apple leaves. Although quercetin derivatives were not the major polyphenolic component of the apple sample (Table 2 and Table 3), these compounds are very important for maintaining human health. Quercetin was found to inhibit human prostate and lung cancer cell growth [5,31], and to reduce the incidence of cardiovascular diseases [39]. This investigation clearly shows that apple leaves could be a very important raw material for the production of flavonol preparations. 

### 3.3. Antioxidant Capacity Fruits and Leaves of Apple cv. Apple

In this study, the results of the improved ABTS and DPPH methods were expressed as the same unit, i.e., Trolox equivalent antioxidant capacity (TEAC; mM Trolox/g), in order to directly compare the tested results of these two methods. For the apple and leaf extracts, the TEAC values found by us were reported in Table 4. The fruit development influenced the decrease in the antioxidant capacity measured by DPPH from 46.1 mM for the unripe apple (grown 60 days after full bloom) to 13.9 mM for the ripe apple (145 days after bloom), and for ABTS^•+^ this was from 4.8 to 1.4 mM TEAC, respectively, for Ozark Gold cv. The leaves, which were collected at the same time as the fruits, possessed a much higher antioxidant capacity measured by both methods. The antioxidant capacity for the first unripe fruits (60 days after full bloom) was higher for ripe fruits (145 days after bloom). The variation of the antioxidant capacity among the leaf samples collected at different periods was small in comparison to the behavior of the fruits. This result was consistent with the total polyphenolic concentration in the analyzed samples. The differences in TEAC between the apple fruits and leaves could be preliminarily attributed to their different contents of polyphenols (Table 2 and Table 3). Unripe apples collected 60 days after bloom had more total polyphenols than the ripe fruits collected 145 days after bloom and proportionally a higher antioxidant activity as well (Table 4).

These results are in accordance with Shiow et al. [30], who analyzed the fruits and leaves from different cultivars of berry plants for their antioxidant capacity by oxygen radicals antioxidant capacity (ORAC) methods and total phenolic content. They reported that blackberries and strawberries had the highest antioxidant potential during the green stages rather than when fully ripened. They also characterized that leaves from these plants have higher antioxidant capacities and total phenolic contents when compared to their fruit tissues. Additionally, they suggested that when the leaves became older, the total phenolic contents and antioxidant power decreased [30]. Liaudanskas et al. [38] postulated that a higher antioxidant activity (evaluated by different methods as ABTS, DPPH, FRAP) of leaves is the result of a high content of some phenolic compounds, such as dihydrochalcones and quercetin glycosides.

## 4. Conclusions

Our results confirmed that the concentrations of polyphenols were highest at the first stage of maturation in fruits and leaves, with the mean concentrations of polyphenols corresponding to much higher antioxidant capacity. The concentrations of dihydrochalcones, flavanols, quercetin derivatives and antioxidant activity were higher in the leaves than in the fruits. This investigation clearly shows that apple leaves could be a very important raw material for the production of polyphenol preparations. All this information may be useful for the promotion of the use of *Malus domestica* unripe fruits and young leaves’ extracts as natural antioxidants in food with beneficial health properties and medicinal products. However, the content of bioactive compounds, such as those present in fruits, depends on cultivars. Moreover, the changes of phenolic composition are strictly related to climatic conditions, especially temperature, irradiation and water availability; therefore, this study needs more replications in the coming years before any conclusions can be drawn on this topic.

Experimental results proved that thinning apples and leaves can be considered a good source of bioactive compounds, which are clearly reflected in the high values of antioxidant properties.

## Figures and Tables

**Table 1:** Characterization of the phenolic compounds of apple fruits by using their spectral characteristics in LC–diode array detector (DAD) (Retention Time λ_max_) negative ions in HPLC–MS.

Group of Polyphenols	R_t_ (min)	λ_max_ (nm)	Compound	Molecular Weight	MS (M−H)^−^	MS/MS Fragments
hydroxycinnamic acid	19.1	305	*p*-coumarylquinic acid	338	337	191
	19.9	320	5-*O*-caffeoylquinic acid	354	353	191
	23.5	305	caffeic acid hexose conjugate	342	341	179
flavanol and procyanidins	16.8	275	procyanidin B1	578	577	289/245
	19.5	280	(+)-catechin	290	289	245
	20.8	275	procyanidin B2	578	577	289/245
	22.4	275	(−)-epicatechin	290	289	245
	24.5	280	procyanidin C1	866	865	577/289/245
dihydrochalcones	34.0	285	phloretin-2′-*O*-xyloglucoside	568	567	273
	36.8	285	phloretin-2′-*O*-glucoside	436	435	273
flavonols	31.6	350	quercetin- 3-*O*-rutinoside	610	609	301
	32.4	355	quercetin-3-*O*-galactoside	464	463	301
	32.9	350	quercetin-3-*O*-glucoside	464	463	301
	34.3	355	quercitin-3-*O*-arabinoside	434	433	301
	35.5	350	quercitin-3-*O*-xyloside	434	433	301
	36.4	345	quercetin-3-*O*-rhamnoside	448	447	301

**Table 2:** Change of the polyphenol content (g kg^−1^ dw) in the different apple cvs. during the developing and ripening of the fruits.

Phenolic Compounds	Cultivars											
	Ozark Gold				Starkinson				Kosztela			
	Fruits after Full Bloom (Days)											
	60	80	130	145	60	80	130	145	60	80	130	145
CQA	0.88 ± 0.18 ^d,^*	0.69 ± 0.03 ^c,d^	0.37 ± 0.02 ^d^	0.27 ± 0.08 ^e^	0.13 ± 0.02 ^c^	0.09 ± 0.03 ^c^	0.05 ± 0.01 ^c^	0.04 ± 0.01 ^c^	6.33 ± 0.08 ^b^	4.43 ± 0.06 ^b^	2.17 ± 0.04 ^b^	1.62 ± 0.03 ^b^
CGLU	0.17 ± 0.05 ^k^	0.12 ± 0.02 ^n,m^	0.04 ± 0.03 ^l,m^	0.03 ± 0.01 ^n^	0.02 ± 0.00 ^e^	0.02 ± 0.01 ^d^	0.04 ± 0.00 ^e^	0.05 ± 0.00 ^e^	nd	nd	nd	nd
PCQ	0.12 ± 0.01l	0.12 ± 0.01 ^n^	0.08 ± 0.04 ^k^	0.04 ± 0.02 ^m^	0.02 ± 0.01 ^e^	0.01 ± 0.00 ^d^	0.01 ± 0.00 ^d^	0.01 ± 0.00 ^d^	0.17 ± 0.02 ^e^	0.12 ± 0.02 ^f^	0.05 ± 0.01 ^f^	0.02 ± 0.00 ^f^
CAd	0.06 ± 0.00 ^p^	0.06 ± 0.02 ^p^	0.02 ± 0.00 ^o^	0.03 ± 0.01 ^o^	0.00 ± 0.00 ^e^	0.00 ± 0.00 ^e^	0.00 ± 0.00 ^e^	0.00 ± 0.00 ^e^	0.12 ± 0.00 ^e^	0.08 ± 0.00 ^g^	0.01 ± 0.00 ^g^	0.01 ± 0.00 ^f^
EC	1.08 ± 0.02 ^c^	0.70 ± 0.02 ^c^	0.41 ± 0.03 ^c^	0.36 ± 0.01 ^c^	0.16 ± 0.02 ^b^	0.09 ± 0.03 ^c^	0.05 ± 0.01 ^c^	0.05 ± 0.01 ^c^	2.35 ± 0.04 ^c^	1.65 ± 0.04 ^c^	0.61 ± 0.02 ^c^	0.60 ± 0.02 ^c^
PB2	1.16 ± 0.34 ^b^	1.08 ± 0.04 ^b^	0.62 ± 0.04 ^b^	0.51 ± 0.01 ^b^	0.17 ± 0.01 ^b^	0.14 ± 0.02 ^b^	0.08 ± 0.01 ^b^	0.07 ± 0.01 ^b^	0.30 ± 0.00 ^e^	0.21 ± 0.01 ^e^	0.11 ± 0.00 ^e^	0.66 ± 0.03 ^c^
PC1	0.84 ± 0.15 ^e^	0.58 ± 0.02 ^f^	0.40 ± 0.05 ^c^	0.27 ± 0.05 ^d^	0.12 ± 0.02 ^c^	0.07 ± 0.01 ^c^	0.05 ± 0.01 ^c^	0.03 ± 0.00 ^c^	0.81 ± 0.04 ^d^	0.57 ± 0.01 ^d^	0.61 ± 0.01 ^c^	0.35 ± 0.02 ^d^
CAT	0.33 ± 0.01 ^g^	0.42 ± 0.03 ^h^	0.14 ± 0.02 ^g^	0.07 ± 0.00 ^k^	0.05 ± 0.01 ^e^	0.05 ± 0.01 ^d^	0.02 ± 0.00 ^d^	0.01 ± 0.00 ^d^	0.76 ± 0.03 ^d^	0.53 ± 0.01 ^d^	0.13 ± 0.02 ^d^	0.08 ± 0.00 ^f^
PB1	0.54 ± 0.04 ^e^	0.62 ± 0.03 ^e^	0.25 ± 0.04 ^e^	0.20 ± 0.02 ^g^	0.08 ± 0.01 ^d^	0.08 ± 0.01 ^c^	0.03 ± 0.00 ^c^	0.03 ± 0.00 ^c^	0.41 ± 0.03 ^d^	0.28 ± 0.01 ^e^	0.09 ± 0.02 ^e^	0.18 ± 0.02 ^e^
PLXG	0.29 ± 0.02 ^i^	0.24 ± 0.03 ^j^	0.12 ± 0.03 ^i^	0.10 ± 0.03 ^ij^	0.00 ± 0.01 ^f^	0.00 ± 0.00 ^e^	0.00 ± 0.00 ^e^	0.00 ± 0.00 ^e^	0.00 ± 0.00 ^g^	0.00 ± 0.00 ^h^	0.00 ± 0.00 ^h^	0.00 ± 0.00 ^g^
PLG	0.42 ± 0.02 ^f^	0.35 ± 0.02 ^i^	0.14 ± 0.05 ^f^	0.13 ± 0.01 ^h^	0.06 ± 0.01 ^d^	0.04 ± 0.01 ^d^	0.02 ± 0.00 ^d^	0.02 ± 0.00 ^c^	0.41 ± 0.01 ^d^	0.29 ± 0.01 ^e^	0.20 ± 0.01 ^e^	0.17 ± 0.00 ^e^
QRUT	nd	nd	nd	nd	nd	nd	nd	nd	nd	nd	nd	nd
QGAL	0.31 ± 0.01 ^h^	0.50 ± 0.03 ^g^	0.13 ± 0.02 ^h^	0.21 ± 0.02 ^f^	0.05 ± 0.01 ^d^	0.06 ± 0.01 ^c^	0.02 ± 0.00 ^d^	0.03 ± 0.01 ^c^	0.58 ± 0.03 ^d^	0.41 ± 0.00 ^d^	0.11 ± 0.02 ^e^	0.13 ± 0.00 ^e^
QGLU	0.05 ± 0.00 ^n^	0.14 ± 0.03 ^l^	0.02 ± 0.00 ^n^	0.04 ± 0.01 ^o^	0.01 ± 0.00 ^e^	0.02 ± 0.01 ^d^	0.00 ± 0.00 ^e^	0.01 ± 0.00 ^d^	0.02 ± 0.00 ^f^	0.02 ± 0.00 ^g^	0.02 ± 0.00 ^f^	0.01 ± 0.00 ^f^
QARA	0.13 ± 0.00 ^l^	0.11 ± 0.01 ^n^	0.04 ± 0.00 ^l^	0.05 ± 0.01 ^l^	0.02 ± 0.01 ^e^	0.01 ± 0.00 ^d^	0.01 ± 0.00 ^d^	0.01 ± 0.00 ^d^	0.07 ± 0.00 ^f^	0.05 ± 0.00 ^g^	0.06 ± 0.00 ^f^	0.06 ± 0.00 ^f^
QXYL	0.24 ± 0.00 ^j^	0.22 ± 0.04 ^j,k^	0.09 ± 0.01 ^j^	0.11 ± 0.04 ^i^	0.04 ± 0.01 ^e^	0.03 ± 0.00 ^d^	0.01 ± 0.00 ^d^	0.01 ± 0.00 ^d^	0.17 ± 0.00 ^e^	0.12 ± 0.02 ^f^	0.09 ± 0.02 ^e^	0.04 ± 0.00 ^f^
QRHM	0.11 ± 0.02 ^m^	0.09 ± 0.03 ^o^	0.03 ± 0.00 ^n^	0.04 ± 0.01 ^m^	0.02 ± 0.00 ^e^	0.01 ± 0.00 ^d^	0.00 ± 0.00 ^e^	0.01 ± 0.00 ^d^	nd	nd	nd	nd
PO	32.43 ± 1.6 ^a^	28.49 ± 5.14 ^a^	12.47 ± 3.34 ^a^	11.80 ± 2.11 ^a^	5.01 ± 0.06 ^a^	3.87 ± 0.10 ^a^	1.65 ± 0.06 ^a^	1.68 ± 0.16 ^a^	20.79 ± 1.11 ^a^	14.55 ± 1.21 ^a^	8.19 ± 0.07 ^a^	5.30 ± 0.05 ^a^
DP	4.57	4.42	4.99	4.19	4.57	4.27	4.99	4.19	5.72	5.55	5.72	4.05
Total	39.15	34.46	15.37	14.22	5.97	4.62	2.05	2.05	33.39	23.37	12.54	9.31

nd—not detected; CQA—chlorogenic acid; CGLU—cyanidin 3-*O*-glucoside; PCQ—*p*-coumaroyloquinic acid; CAd—caffeic acid hexose conjugate; EC—(−)-epicatechin; PB2—procyanidins B2; PC1—procyanidins C1; CAT—(+)-catechin; PB1—procyanidins B1; PLXG—phloretin 2′-xyloglucose; PLG—phloretin 2′-glucose; QRUT—quercetin-3-O-rutinoside; QGAL—quercetin-3-*O*-galactoside; QGLU—quercetin-*O*-3-glucoside; QARA—quercetin-3-*O*-arabinoside; QXYL—quercetin-3-*O*-xyloside; QRHM—quercetin-3-*O*-rhamnoside; PO—polymeric procyanidins; DP—degree of polymerization; * values are the means ± standard deviation *n* = 3; the mean values within a verse with different letters are significantly different at *p* < 0.05.

**Table 3:** Change in the polyphenol content (g kg^−1^ dw) in the leaves of Ozark Gold cv. during the developing and ripening of the fruit.

Phenolic Compounds	Leaves after Full Bloom (Days)			
	60	80	130	145
CQA	1.17 ± 0.12 ^i,^*	1.13 ± 0.24 ^i^	1.46 ± 0.12 ^h^	16.6 ± 1.50 ^g^
CGLU	nd	nd	nd	nd
PCQ	0.19 ± 0.01 ^n^	0.25 ± 0.05 ^m^	0.22 ± 0.03 ^l^	0.22 ± 0.04 ^l^
CAd	0.20 ± 0.05 ^m^	0.24 ± 0.09 ^m^	0.20 ± 0.05 ^l,m^	0.19 ± 0.02 ^m^
EC	0.62 ± 0.07 ^j^	0.74 ± 0.06 ^j^	0.85 ± 0.11 ^j^	0.96 ± 0.04 ^h^
PB2	0.20 ± 0.09 ^m^	0.20 ± 0.01 o	0.22 ± 0.04 ^l^	0.29 ± 0.03 ^k^
PC1	0.12 ± 0.01 ^p^	0.11 ± 0.03 ^p^	0.14 ± 0.01 ^n^	0.17 ± 0.01 ^n^
CAT	0.07 ± 0.05 ^r^	0.06 ± 0.02 ^r^	0.05 ± 0.00 ^o^	0.06 ± 0.01 ^p^
PB1	0.05 ± 0.02 ^s^	0.05 ± 0.00 ^s^	0.04 ± 0.01 ^p^	0.05 ± 0.04 ^s^
PLXG	5.42 ± 0.16 ^f^	6.24 ± 0.18 ^c^	3.27 ± 0.34 ^f^	3.39 ± 0.03 ^f^
PLG	97.05 ± 3.34 ^a^	110.15 ± 2.43 ^a^	110.75 ± 1.16 ^a^	109.70 ± 2.57 ^a^
QRUT	0.13 ± 0.04 ^o^	0.69 ± 0.11 ^l^	0.60 ± 0.19 ^i^	0.76 ± 0.02 ^i^
QGAL	7.36 ± 0.13 ^c^	6.05 ± 0.35 ^d^	4.33 ± 0.23 ^c^	5.45 ± 0.18 ^c^
QGLU	6.77 ± 1.61 ^d^	5.66 ± 1.38 ^e^	3.87 ± 0.17 ^d^	4.75 ± 1.35 ^d^
QARA	3.51 ± 1.23 ^g^	3.93 ± 0.34 ^g^	3.74 ± 0.34 ^e^	3.80 ± 1.02 ^f^
QXYL	2.30 ± 0.91 ^h^	2.58 ± 0.68 ^h^	2.17 ± 0.21 ^g^	2.43 ± 0.24 ^g^
QRHM	5.52 ± 1.28 ^e^	4.86 ± 0.49 ^f^	3.85 ± 1.34 ^d^	4.18 ± 1.18 ^e^
PO	19.06 ± 2.16 ^b^	17.55 ± 1.16 ^b^	17.09 ± 2.51 ^b^	22.15 ± 3.48 ^b^
DP	9.41	10.06	9.18	8.42
Total	150.20	160.93	153.15	160.65

nd—not detected; CQA—chlorogenic acid; CGLU—cyanidin 3-glucoside; PCQ—*p*-coumaroyloquinic acid; CAd—caffeic acid hexose conjugate; EC—(−)-epicatechin; P B2—procyanidins B2; PC1—procyanidins C1; CAT—(+)-catechin; PB1—procyanidins B1; PLXG—phloretin 2′-xyloglucose; PLG—phloretin 2′-glucose; QRUT—quercetin-3-O-rutinoside; QGAL—quercetin-3-*O*-galactoside; QGLU—quercetin-*O*-3-glucoside; QARA—quercetin-3-*O*-arabinoside; QXYL—quercetin-3-*O*-xyloside; QRHM—quercetin-3-*O*-rhamnoside; PO—polymeric procyanidins; DP—degree of polymerization; * values are the means ± standard deviation *n* = 3; the mean values within a verse with different letters are significantly different at *p* < 0.05.

**Table 4:** Change in the antioxidant capacity (mM Trolox/g dw) during the developing and ripening of the fruits and leaves.

Days after Full Bloom	DPPH				ABTS			
	Ozark Gold		Starkinson	Kosztela	Ozark Gold		Starkinson	Kosztela
	Leaves	Fruits			Leaves	Fruits		
60	164.7 ± 2.9 ^a,^*	46.1 ± 3.1 ^a^	20.1 ± 1.6 ^a^	43.9 ± 3.4 ^a^	17.5 ± 1.6 ^c^	4.8 ± 0.5 ^a^	2.6 ± 0.6 ^a^	4.5 ± 0.5 ^a^
80	158.3 ± 1.7 ^c^	31.1 ± 1.0 ^b^	11.7 ± 2.1 ^b^	30.4 ± 1.2 ^b^	19.9 ± 2.5 ^a^	4.3 ± 0.4 ^b^	2.0 ± 0.3 ^b^	4.0 ± 0.3 ^a^
130	153.0 ± 1.8 ^d^	14.4 ± 1.5 ^c^	8.8 ± 0.6 ^c^	15.7 ± 1.9 ^c^	18.5 ± 0.9 ^b^	1.4 ± 0.2 ^c^	1.5 ± 0.5 ^c^	2.6 ± 0.2 ^b^
145	146.7 ± 0.9 ^b^	13.9 ± 1.6 ^d^	3.6 ± 0.9 ^d^	11.7 ± 0.4 ^d^	18.7 ± 1.7 ^b^	1.4 ± 0.1 ^c^	0.9 ± 0.2 ^d^	1.4 ± 0.3 ^c^

* Values are the means ± standard deviation *n* = 3; the mean values with different letters are significantly different at *p* < 0.05.
